# Bridging the gap in antioxidant activity of flavonoids: Correlating the oxidation of human plasma with chemical and cellular assays

**DOI:** 10.1016/j.crfs.2024.100714

**Published:** 2024-03-13

**Authors:** Nima Mohammadi, Amanda dos Santos Lima, Luciana Azevedo, Daniel Granato

**Affiliations:** a Bioactivity & Applications Lab, Department of Biological Sciences, Faculty of Science and Engineering, University of Limerick, V94 T9PX, Limerick, Ireland; b Laboratory of Nutritional and Toxicological Analyses in vitro and in vivo (LANTIN), Federal University of Alfenas, Alfenas-MG, Brazil; c Bernal Institute. University of Limerick, V94 T9PX, Limerick, Ireland

**Keywords:** Cytotoxicity, Reactive oxygen species, Flavonoids, Structure-activity relationship, Interaction effect

## Abstract

Traditional antioxidant screening relies on chemical assays to assess compounds' potential in combating oxidative processes. However, translating chemical antioxidant activity to complex biological systems poses challenges. In this study, the antioxidant potential of fruit-derived phenolic compounds, hyperoside (HP), epicatechin (EC), and phlorizin (PZ), and their combinations in a specific ratio were investigated using a simplex-centroid design of experiments. The research included *in vitro* antioxidant assays, plasma protection against oxidation tests, and cytotoxicity assessments in human cell lines. The results revealed the complex relationship between chemical antioxidant activity and its relevance to cellular oxidative and antioxidative processes. HP and EC exhibited significant antioxidant activity, with HP outperforming EC in multiple assessments. Cytotoxicity assay confirmed that these compounds did not induce cell death or hinder proliferation, even at higher concentrations (>100 μmol/mL). In the cell antioxidant activity (CAA) test, HP and EC exhibited higher CAA, while PZ displayed lower antioxidant activity. In conclusion, a synergistic effect emerged when HP, EC, and PZ were combined, particularly in plasma protection, suggesting protective effects and potential health benefits. This research emphasized the need for a nuanced understanding of the interplay between chemical assays and cellular behavior in comprehending the relationship between chemical-based, human plasma oxidation, and CAA.

## Highlights

• Hyperoside excels in multiple assays, showing superior antioxidant activity.
• Phlorizin stands out in superoxide radical scavenging activity, complementing hyperoside ‘s strengths.
• Combined, hyperoside, epicatechin, and phlorizin enhance plasma protection against oxidative stress.
• Hyperoside and epicatechin mitigate H_2_O_2_-induced stress in human cells.
• Integrating chemical and biological assays is crucial for antioxidant evaluation.

## Graphical abstract

**Figure:**
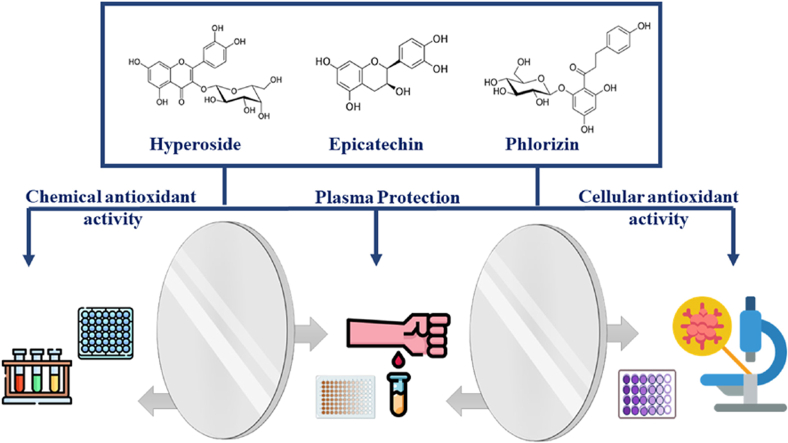

## 1 Introduction

Dietary flavonoids are a subclass of phenolic compounds, encompassing substances like hyperoside (HP), epicatechin (EC), and phlorizin (PZ). These phenolic constituents are commonly present in a variety of fruits and vegetables. Apple pomace, a byproduct of apple processing, yields about 4 million tons of waste yearly. Apple pomace contains various phenolic compounds, including HP, EC, PZ, chlorogenic acid, catechin, syringic acid, *p*-coumaric acid, ferulic acid, and quercetin (Mohammadi et al., 2024). Among these polyphenols, HP, EC, and PZ are the primary polyphenols in apple pomace extracts. HP falls under the category of flavonoid glycosides, consisting of a flavonoid molecule (aglycone) bonded to a sugar molecule (glycone). HP contains three notable functional groups in its structure: a hydroxyl group (-OH) and a carbonyl group (C=O) within its sugar moiety. Significantly, its hydroxyl groups play a crucial role in its antioxidative and free radical scavenging properties. HP has various pharmacological effects, including its role in cancer prevention and organ protection (Xu et al., 2022). EC belongs to the flavanol group and possesses a unique chemical structure defined by a catechol moiety (Uchida et al., 2016). EC's antioxidative properties originate from its catechol moiety, which consists of two adjacent hydroxyl groups. The antioxidant activity of EC is primarily attributed to the hydrogen bond interaction at the catechol moiety (Leopoldini et al., 2011). The antioxidant capability of ECT is significantly affected by the absence of the C2=C3 double bond in the C ring (Vagánek et al., 2014). Moreover, it has been demonstrated that the reactivity of flavan-3-ols undergoes further thermodynamic alterations by the influence of solvents (Chen et al., 2015; Xue et al., 2013). PZ, characterized by a molecular structure referred to as dihydrochalcone, represents a flavonoid variant. Phloretin in PZ displays low bond dissociation enthalpies (BDEs) in relation to its hydroxyl (OH) groups, indicating its capability to donate hydrogen atoms and counteract free radicals. As a result, the phloretin component plays a crucial role in the antioxidant activity of PZ (Mendes et al., 2018). Nevertheless, the antioxidant activity and the interactions among HP, EC, and PZ remain to be fully understood not only in a model solution (e.g., as carried out herein) or in complex matrices, such as apple juices. A study conducted by Rezk et al. (2002) revealed that both phloretin and PZ exhibit antioxidant capabilities (Rezk et al., 2002). Notably, phloretin displayed superior efficacy compared to PZ in terms of antioxidant capacity and network pharmacology (Ongay et al., 2023). Additionally, it is reported that the antioxidant activity of phloretin, an aglycon flavonoid, decreases upon α-glucosylation. However, this decrease is moderate, and the activity can be restored upon *in vivo* deglycosylation (Gonzalez-Alfonso et al., 2021).

Food synergy highlights that the combined effects of various minor components, such as phenolic compounds, in whole foods exert a more substantial influence on health than the study of individual substances alone (Jacobs and Temple, 2012). This concept is crucial in understanding and addressing nutrition-related health conditions, aiming to optimize mitigation nutritional-based strategies. Despite progress in understanding individual compounds, the interactions among various phenolic compounds remain partially understood. Research investigating combinations of phenolic compounds remains relatively constrained; however, researchers reported that interaction between quercetin and naringenin, two common flavonoids, significantly impacted their electrochemical properties and redox-related bioactivities. Despite being weak antioxidants, their combined effect showed synergy, amplifying their reducing activity. In cellular tests, the mixtures stimulated cell growth, challenging the idea that isolated flavonoids produce a similar effect (Baranowska et al., 2021). The research discovered that the combinations of *p*-coumaric with ferulic acids and caffeic with sinapic acids had the highest synergistic effects among hydroxycinnamic acids. The overall antioxidant activity was influenced by factors such as compound concentration, the number and position of functional groups, and other variables, including intramolecular hydrogen bonds, dissociation, and electron effects (Skroza et al., 2022). Our previous study highlighted positive interactions among catechins, enhancing both chemical and cell-based antioxidant activities while concurrently diminishing cytotoxicity (Xu et al., 2021). Moreover, this research indicated that optimal combinations of flavanols elevated anti-proliferative activity and reduced the generation of intracellular reactive oxygen species. Previous research demonstrated synergistic effects from combinations of phenolic acids. This decreased the minimum inhibitory concentration against *Staphylococcus aureus* (Cui et al., 2019). Furthermore, interactions between coffee phenolics and other phenols can result in either synergistic or antagonistic effects on diverse bioactive properties, including chelating power, enzyme inhibition, and antioxidants (Erskine et al., 2022). Synergistic effects were noted between rosmarinic acid and quercetin, as well as between rosmarinic acid and caffeic acid. Conversely, antagonistic effects were observed in combinations of α-tocopherol/caffeic acid, tocopherol/rosmarinic acid, catechin/caffeic acid, and caffeic acid/quercetin (Peyrat-Maillard et al., 2003).

Currently, there is a lack of research investigating the statistical approach to the individual, binary, and trinary combinations of HP, EC, and PZ. This research gap underscores the importance of exploring the mechanisms of action and inherent interactions within these compounds. Obtaining insights into phenomena such as antagonism and synergism effects has the potential to shed light on how these compounds counteract induced oxidation within human cells and plasma. This foundation provides the groundwork for the potential creation of innovative nutraceuticals, a venture pursued by both the food and pharmaceutical sectors, aiming to deliver bioactive agents like HP, EC, and PZ.

Investigating phenolic compounds' antioxidant and cytotoxic effects and their potential effects in human blood is critical in comprehending their biological implications. Examining the interplay between chemical *in vitro* assays, cell-based ROS generation in human cells, and the protective effects against lipoperoxidation in human plasma attempts to illuminate the effectiveness and relevance of existing antioxidant screening methods. The question arises as to whether these methods need to be reevaluated or adapted in the context of complex biological systems. Furthermore, the broader context of public health objectives and United Nations Sustainable Development Goal 3, which seeks to advance good health and well-being, is embraced. As this research is embarked upon, specific objectives are aimed: (i) Assess synergistic and antagonistic effects of HP, EC, and PZ, and their binary/ternary mixtures, through chemical antioxidant assays; (ii) Investigate the cellular antioxidant activity (CAA) in human cell lines and plasma and (iii) Examine any correlations between chemical antioxidant activity, plasma protection against oxidation, and CAA better to understand the complex relationship between different analytical approaches.

## 2 Materials and methods

### 2.1 Chemicals and cell lines

Gallic and ascorbic acids, 2 N, 2,2-diphenyl-1-picrylhydrazyl (DPPH) radical, neocuproin, 1,10-phenanthroline, Iron (II) sulfate heptahydrate (FeSO_4_·7H_2_O), ammonium acetate (NH_4_CH_3_CO_2_), copper (II) chloride dihydrate (CuCl_2_·2H_2_O), tris-HCl, pyrogallol, sodium phosphate monobasic monohydrate (NaH_2_PO_4_·H_2_O), sodium (mono)hydrogen phosphate (Na_2_HPO_4_·6H_2_O), sodium chloride (NaCl), penicillin,dichloro-dihydro-fluorescein diacetate (DCFH-DA), 3–4,5 dimethylthiazol-2, diphenyl tetrazolium bromide (MTT), Dulbecco's Modified Eagle's Medium/Nutrient Mixture F-12 Ham (DMEM)were obtained from Sigma-Aldrich (Darmstadt, Germany). HP, EC, and PZ were obtained from Extrasynthese (Genay, France). Aqueous solutions were prepared using ultrapure water (Millipore, São Paulo, Brazil). A549 (lung adenocarcinoma epithelial cells), HepG2 cell lines (human hepatoma carcinoma cells), HUVEC (normal primary Human Umbilical Vein Endothelial Cells), and HCT8 (human ileocecal adenocarcinoma cells) were purchased from Rio de Janeiro Cell Bank (Rio de Janeiro, Brazil).

### 2.2 Experimental design

A simplex-centroid experimental design with additional points (totaling 10 combinations) was employed to investigate the influence of HP, EC, and PZ on the chemical antioxidant capacity, as presented in Table 1. To achieve this objective, single compounds (HP, EC, and PZ), binary mixtures (3 combinations), and ternary mixtures (4 combinations) were evaluated. This experimental arrangement identifies potential synergistic, antagonistic, and additional effects between phenolic compounds. To ensure a proper molar basis for comparison, each phenolic compound was tested at 1 mmol/L using methanol as the solvent. After the preparation of each solution, the measurement of antioxidant capacity was conducted in a randomized order to prevent any carryover effects.

**Table 1:** The effects of hyperoside (HP), epicatechin (EC), and phlorizin (PZ) and their binary/ternary combinations on FCRC, CUPRAC, HRSA, DPPH, SRSA, plasma protection against induced oxidation, CAA.

Assay	HP	EC	PZ	FCRC (mg GAE/L)	CUPRAC (AAE mg/L)	HRSA (mg GAE/L)	DPPH (AAE mg/L)	SRSA (AAE mg/L)	Plasma protection (AAE mg/L)	CAA (% ROS generation)
1	1	0	0	314±6^ab^	1316 ± 40^a^	193±2^a^	385 ± 23^a^	502 ± 16^b^	1022 ± 91^a^	35±5^b^
2	0	1	0	238 ± 12^bcd^	1230 ± 15^b^	37.68^g^	214 ± 13^c^	483 ± 49^b^	230 ± 53^f^	23±0^bcd^
3	0	0	1	166±2^d^	110±4^g^	5±0^i^	26±1^f^	955 ± 87^a^	821 ± 25^b^	161 ± 13^a^
4	0.5	0.5	0	314 ± 39^a^	1075±7^c^	137±1^c^	271 ± 14^b^	599±3^b^	218 ± 36^f^	16±2^cde^
5	0.5	0	0.5	223 ± 10^cd^	746±6^e^	103±1^d^	163±6^d^	478 ± 63^b^	519 ± 36^d^	25±2^bc^
6	0	0.5	0.5	177 ± 21^d^	684 ± 11^e^	26±1^h^	127±2^e^	463 ± 77^b^	327 ± 81^ef^	6±0^de^
7	0.33	0.33	0.34	230 ± 10^bcd^	853 ± 12^d^	97±1^e^	168±8^d^	571 ± 44^b^	595 ± 25^c^	6±0^de^
8	0.67	0.16	0.17	253 ± 12^abc^	1207±8^b^	155±1^b^	220 ± 10^c^	457±9^b^	427 ± 58^de^	4±1^e^
9	0.17	0.67	0.16	231 ± 14^cd^	1061 ± 14^c^	46±1^f^	213 ± 13^c^	430 ± 51^b^	653 ± 15^bc^	2±0^e^
10	0.17	0.16	0.67	182 ± 10^d^	551±7^f^	38±0^g^	102±3^e^	405±9^b^	704 ± 29^bc^	2±0^e^

Note: HP = Hyperoside; EC = Epicatechin; PZ = Phlorizin; FCRC = Folin-Ciocalteu reducing capacity; CUPRAC = cupric ion reducing antioxidant capacity; HRSA = hydroxyl radical scavenging activity; DPPH = free-radical scavenging activity; SRSA = superoxide radical scavenging activity; GAE = gallic acid equivalents; AAE = ascorbic acid equivalents; CAA = cellular antioxidant activity; ROS = reactive oxygen species. Different superscript letters in the same column represent statistically different results (p < 0.05).

### 2.3 *In vitro* antioxidant activity

The samples' Folin-Ciocalteau reduction capacity (FCRC) was evaluated using a colorimetric method based on the procedure described by Fidelis et al. (2018). The results were expressed as mg of gallic acid equivalents per liter (mg GAE/L). Cupric-ion reducing antioxidant capacity (CUPRAC) was evaluated following the methodology outlined by Corrigan et al. (2023). The results were presented as mg of ascorbic acid equivalents per liter (mg AAE/L). The free-radical scavenging activity toward the DPPH radical was performed using a DPPH concentration of 0.10 mmol/L in methanol, and the data were expressed as mg AAE/L (Corrigan et al., 2023). Hydroxyl radical scavenging activity (HRSA) was determined using the 1,10-phenanthroline-H_2_O_2_ spectrophotometric method, and the results were expressed as mg GAE/L (Corrigan et al., 2023). The superoxide radical scavenging activity (SRSA) was conducted based on the method outlined by Zhang et al. (2016), with some adjustments. In each well, 30 μL of diluted sample or water (blank) and 135 μL of Tris-HCl buffer (50 mmol/L, pH 8.2, containing Na_2_EDTA at 2 mmol/L) were added. Following a 10-min incubation in the dark, 30 μL of pyrogallol at 2.5 mmol/L, prepared in 1 mM HCl, was added to each well. After 15 min, the absorbance was measured at 320 nm All analyses were performed thrice. The scavenging activity was determined using Eq. (1), and the data were expressed as mg AAE/L.

(1) $$\text{Scavenging}\;\text{activity}\;\left(\%\right)=\left[1\;–\;\left(A_{1}/\;A_{0}\right)\right]\times 100$$

Where A_0_ and A_1_ are the absorbance of water and sample mixture, respectively. All experiments were conducted at 25 °C.

### 2.4 Antioxidant activity in human plasma

Blood was collected from a healthy female volunteer (30 years old, BMI< 25 kg/m^2^). The study was carried out in accordance with the Ethical Committee of the University of Limerick's guidelines, under approval number 2023_02_01_S&E. Plasma was isolated from red blood cells (RBCs) using centrifugation at 900 *g* for 5 min. The collected plasma was diluted 40-fold with PBS and stored at 4 °C until it was ready for analysis (Mohammadi et al., 2024). The plasma oxidation assay followed the protocol established by Mohammadi et al. (2024). Briefly, in a 96-well UV plate, a sample in PBS was mixed with plasma. After a 15-min incubation at 37 °C, lipid oxidation was induced by adding CuCl_2_ in PBS. Following a 2-h incubation at 37 °C, a microplate reader monitored the production of conjugated dienes at 3-min intervals at 245 nm. The results were reported as mg AAE/L.

### 2.5 Cellular antioxidant activity

The cells were cultured in Dulbecco's Modified Eagle's Medium/Nutrient Mixture F-12 HAM (DMEM), supplemented by heat-inactivated bovine fetal serum (Gibco, EUA) to final concentrations of 10% and 1% of penicillin. These cell cultures were maintained in a humidified incubator at 37 °C with 5% CO_2_. The cytotoxicity, proliferation, and cell death of HP, EC, and PZ and their binary/ternary combinations were evaluated in HepG2, HCT8, HUVEC, and A549 cell lines using the MTT assay. Briefly, the cells are added to a 96-well plate at a confluence of 1 × 10^4^ per well (100 μL). After 24 h of adhesion, the cells were treated with different HP, EC, and PZ concentrations with their combinations (1–100 μmol/mL, 100 μL) for 48 h at 37 °C. Then, 10 μL of MTT (0.5 mg/mL) was added, and after 4 h, DMSO (100 μL) was added to the wells to dissolve the formazan crystals formed by the metabolically active cells. The 50% cell viability inhibition (IC_50_), the 50% growth inhibition (GI_50_), and the 50% cell death (LC_50_)were calculated as outlined by (do Carmo et al., 2018).

To assess the intracellular ROS generation, DCFH-DA was used as a probe. For this experiment, HCT8 cells were used to test different concentrations (10–100 μmol/mL) of HP, EC, and PZ with their binary/ternary combinations. The cells are placed in a 96-well plate (6 × 10^4^ per well) and treated with the samples or 22.5 μM of H_2_O_2_ (positive control) or culture medium (negative control) for 1 h in the dark. Following the treatment, PBS was used to wash the plate, and HANKS solution was added with H_2_O_2_ (22.5 μM). The fluorescence intensity (λ_emission_ = 538 nm and λ_excitation_ = 485 nm) was measured (Escher et al., 2018). The data were expressed as a percentage of fluorescence intensity relative to the untreated group (negative control).

### 2.6 Response surface modeling and statistical analysis

Response Surface Methodology (RSM) was applied to formulate a cubic regression equation (Eq. (2)) using the experimental data, which consisted of triplicate values (n = 40 data points per antioxidant assay). In this equation, the linear (b_i_), quadratic (b_ij_), and cubic (b_ijk_) effects on the response variable (represented as ŷ, i.e., antioxidant activity) were estimated based on triplicate values, resulting in a total of 40 data points for each antioxidant assay.

(2) $$\hat{y}=\sum_{i=1}^{3}b_{i}x_{i}+\sum_{i<j}^{3}\sum_{j}^{3}b_{ij}x_{i}x_{j}+\sum_{i<j}^{3}\sum_{j<k}^{3}\sum_{k}^{3}b_{ijk}x_{i}x_{j}x_{k}$$

Polynomial regression equations were derived using only coefficients that exhibited statistical significance (p < 0.10), and triangular plots were generated to visualize the RSM models. To assess the statistical significance of each model, the determination coefficient (R^2^) and the adjusted R^2^ were estimated using the TIBCO Statistica 13.3 software (TIBCO Ltd, Palo Alto, CA, USA). The relative error, calculated by comparing predicted and observed values, was assessed in relation to the ±95% confidence interval.

The statistical significance was calculated using TIBCO Statistica v.13.3. (Palo Alto, CA, USA) and GraphPad Prism 8 (GraphPad Software, San Diego, CA, USA). For the MTT assay, we performed a nonlinear regression, and for the ROS assay. For all quantitative parameters, one-way ANOVA was performed, followed by Tukey's test (*p* < 0.05). Correlation assessments were conducted using Pearson's correlation coefficients, and significance was attributed to probability values below 0.05.

## 3 Results and discussion

### 3.1 Effects of HP, EC, and PZ on chemical antioxidant activity

The effects of HP, EC, and PZ and their binary/ternary combinations on the reducing capacity (CUPRAC and FCRC), DPPH scavenging activity, and ROS scavenging activity are shown in Table 1. Antioxidants can counteract the harmful effects of reactive species on cell membranes through three primary mechanisms: the ability to chelate transition metals, single electron-proton transfer (SET) and sequential proton-loss electron-transfer (SPLET), and hydrogen atom transfer (HAT) (Granato et al., 2018). Hence, the FCRC, CUPRAC, and DPPH protocols are primarily related to SET, while HRSA and SRSA are associated with the HAT mechanism of action.

HP is a flavonol known as quercetin 3-*O*-β-D-galactopyranoside, which contains four hydroxyl groups whereby two are in *ortho* position on the B ring (C-3′ and C-4’) and two hydroxyl groups in the *meta* position on the A ring (C-5 and C-7) and contains a sugar moiety at C-3 (C ring). HP demonstrated significantly higher (p < 0.05) FCRC, CUPRAC, HRSA, and DPPH, except for SRSA. Our findings indicate that the antioxidant activity of HP is linked to both SET and HAT mechanisms, and its antioxidant activity is higher than that of EC and PZ. Previous research has reported that HP, isolated from *Camellia sasanqua*, exhibited antioxidant activity against DPPH (IC_50_ = 18.3 ± 1.63 μg/mL) (Sukito and Tachibana, 2014). In another study, HP significantly enhanced cell viability, reduced lipid peroxidation, and decreased intracellular ROS in *Saccharomyces cerevisiae* (Gao et al., 2019). In another study, the 80% ethanol extract from the *Rhus coriaria*, containing a high concentration of HP (622.24 mg/kg), exhibited stronger DPPH free radical scavenging activity than the 100% ethanol extract (Caliskan et al., 2022). The antioxidant activity of HP is likely intricately linked to its chemical composition, primarily contingent upon the primary structure and positioning of the galactose sugar moiety attached to this compound. Its effectiveness is impacted by the presence of galactose at position C-3 and the β-hydroxyl groups at C-3′ and C-4′, highlighting the significance of the antioxidant structure–activity relationship (Jang, 2022).

EC, a flavanol that contains four hydroxyl groups whereby two are in *ortho* position (B ring: C-4′ and C-5’) and two hydroxyl groups in *meta* position (A ring: C-5 and C-7), demonstrated modest mean values (p < 0.05) for FCRC, CUPRAC, DPPH, and SRSA, except for HRSA, which had a low value. Flavonoids possessing an *o*-dihydroxy or trihydroxy B ring, such as EC, display potent antioxidant activity via metal chelation, HAT, and SET. The antioxidant action of EC involves oxidation occurring at both the B-ring and A-ring. The oxidation at the B-ring follows a mechanism similar to that observed for peroxyl radicals, whereas oxidation at the A-ring has been observed in the presence of the oxidant system H_2_O_2_ (Sang et al., 2002). In another study, EC exhibited a remarkable IC_50_ value of approximately 1.56 μg/mL for DPPH radical scavenging activity (Jug et al., 2021).

PZ, a dihydrochalcone that contains three hydroxyl groups whereby two are in *meta* position (A ring: C-4′ and C-6’) and one hydroxyl group at C-4 (B ring), demonstrated the lowest (p < 0.05) FCRC, CUPRAC, HRSA, and DPPH, except for SRSA, which had the highest value. According to the data in Tables 1 and it can be observed that PZ exhibited a stronger HAT mechanism in the SRSA assay compared to EC and HP. A previous study reported that PZ showed lower antioxidant activity compared to phloretin when evaluated using three different assays: DPPH, FCRC, and iron-reducing capacity (Ongay et al., 2023). This finding aligns with the results obtained in our current study, where PZ demonstrated lower antioxidant activity compared to the other compounds. PZ contains a β-D-glucopyranosyl residue at C-2′ through a glycosidic linkage, which is susceptible to hydrolysis due to its instability. As a result of this glycosylation, the extent of deprotonation is reduced, leading to a decrease in the ability to scavenge free radicals.

Differences in the chemical structure (Fig. S1) between EC, PZ, and HP are apparent: while EC and HP are flavonoids and contain four hydroxyl groups, PZ is a dihydrochalcone with three hydroxyl groups and does not contain a 3′4′ catechol system. Additionally, both PZ and HP are glycosylated, while EC does not contain any sugar moiety in its structure. The high antioxidant activity of EC and HP can be easily explained by their structure (Fig. S1): both have a catechol group in the B ring, and a hydroxyl group at C-7 (A ring): evidence shows that the presence of a catechol group with hydroxyl groups (OH) in the 3,4-*ortho* position is involved in the antioxidant activity. The radical derived from the H-abstraction of hydroxyl groups at C-3/C-4 can be stabilized by the electron-donating power of the *ortho* hydroxyl and the formation of the intramolecular hydrogen bond (Tejero et al., 2007). Additionally, the 7-O^-^ anion is responsible for the fast kinetics of flavonoid/free radical reaction because HAT (in the B ring) and SPLET (in the A ring) mechanisms occur synchronously (Klein et al., 2016).

**Fig. 1:**
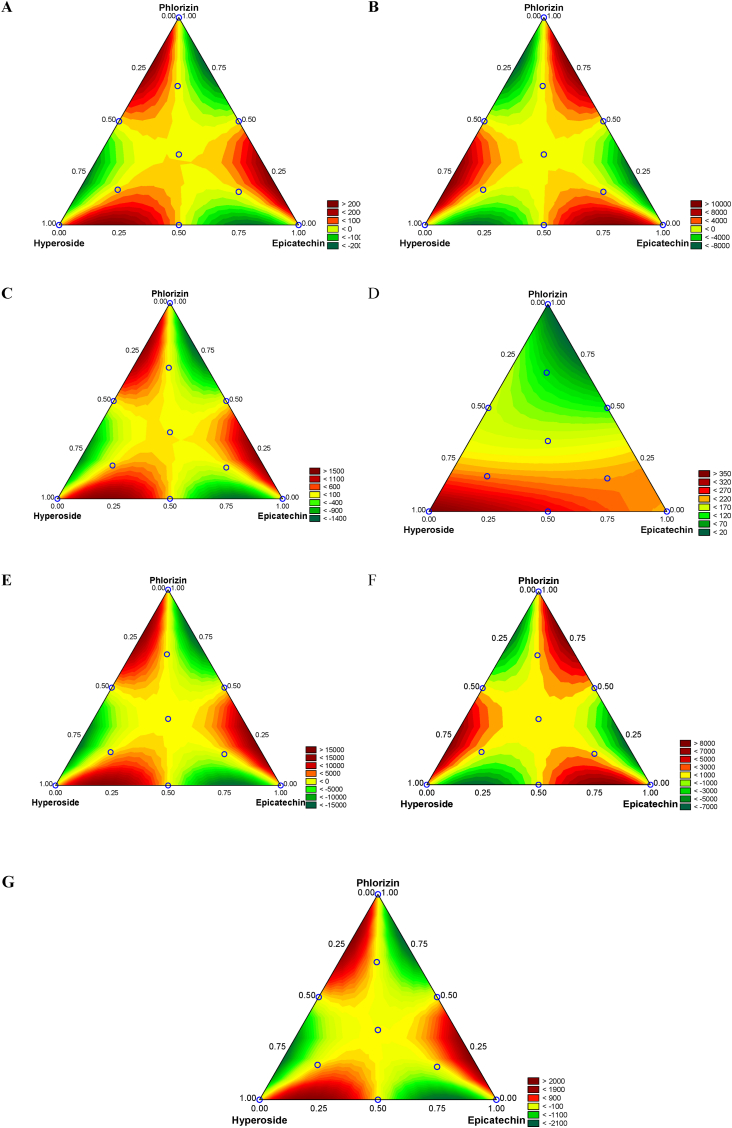
Response surface plots to show the effects of hyperoside (HP), epicatechin (EC), phlorizin (PZ) and their binary and ternary mixtures on Folin-Ciocalteu reducing capacity – FCRC (A), cupric ion reducing antioxidant capacity – CUPRAC (B), hydroxyl radical scavenging activity – HRSA (C), free-radical scavenging activity – DPPH (D), superoxide radical scavenging activity – SRSA (E), plasma protection against oxidation (F), and cellular antioxidant activity (CAA) in HCT8 cells (G).

Using the data from Table 1, RSM was used to evaluate potential interactions (synergism or antagonism) between HP, EC, and PZ on the chemical and cellular antioxidant activity. The results are presented in Table 2 and Fig. 1. For FCRC, HP seemed to exert higher SET compared to EC and PZ, in order. Similarly, the binary interaction HP-EC and EC-PZ were significant (p < 0.10) but did not provide any synergism. These findings are graphically represented in Fig. 1A, where a two-dimensional response plot illustrates varying antioxidant capacity values for all the experimental outcomes. The RSM model accounts for 92.6% of the data variability, showing its suitability for predictive applications.

**Table 2:** Response surface modeling to assess the effects of hyperoside (HP), epicatechin (EC), and phlorizin (PZ) on the chemical antioxidant activity, plasma protection, and cellular antioxidant activity (CAA).

Factors	Regression coefficients	Standard error	*t*-Value	*p*-Value	−95% Confidence limit	+95% Confidence limit
**Folin-Ciocalteu reducing capacity – FCRC**						
(A)HP	308	11	25	<0.001	281	333
(B)EC	237	13	18	<0.001	208	265
(C)PZ	163	11	13	<0.001	136	188
AB	179	60	3	0.011	47	311
BC	−128	60	−2	0.054	−259	3
AB (A-B)	22,936	1132	2	0.065	−1689	47,560
AC (A-C)	−22384	10,961	−2	0.063	−46268	1499
BC(B–C)	23,701	11,648	2	0.064	−1678	49,080
R^2^	0.926					
R^2^_Adj_	0.883					
**Cupric ion reducing antioxidant capacity – CUPRAC**						
(A)HP	1321	13	96	<0.001	1291	1351
(B)EC	1230	12	98	<0.001	1202	1257
(C)PZ	112	12	9	<0.001	85	139
AB	−791	66	−11	<0.001	−937	−645
AC	132	66	2	0.071	−14	277
ABC	1222	452	2	0.020	227	2216
AB (A-B)	−99117	13,247	−7	<0.001	−128,274	−69959
AC (A-C)	97,427	12,852	7	<0.001	69,140	125,714
BC(B–C)	−102744	13,661	−7	<0.001	−132,812	−72676
R^2^	0.998					
R^2^_Adj_	0.997					
**Hydroxyl radical scavenging activity – HRSA**						
(A)HP	192	1	408	<0.001	190	192
(B)EC	38	1	80	<0.001	36	38
(C)PZ	5	1	11	<0.001	4	6
AB	91	2	39	<0.001	86	96
AC	18	2	7	<0.001	12	22
BC	18	2	8	<0.001	13	23
ABC	200	16	12	<0.001	163	237
AB (A-B)	15,577	454	34	<0.001	14,564	16,588
AC (A-C)	−14817	440	−33	<0.001	−15799	−13835
BC(B–C)	15,771	468	33	<0.001	14,727	16,814
R^2^	0.999					
R^2^_Adj_	0.999					
**Free-radical scavenging activity – DPPH**						
(A)HP	371	4	77	<0.001	360	381
(B)EC	213	4	49	<0.001	203	222
(C)PZ	28	4	6	<0.001	18	37
AB	−58	23	−2	0.030	−108	−8
AC	−141	23	−6	<0.001	−191	−90
ABC	−251	141	−1	0.100	−559	56
AC (A-C)	−609	75	−8	<0.001	−774	−445
BC(B–C)	473	79	5	<0.001	300	646
R^2^	0.996					
R^2^_Adj_	0.994					
**Superoxide radical scavenging activity - SRSA**						
(A)HP	502	3	14	<0.001	423	579
(B)EC	483	35	13	<0.001	405	561
(C)PZ	955	35	27	<0.001	877	1033
AB	427	171	2	0.032	44	809
AC	−1000	171	−5	<0.001	−1383	−617
BC	−1022	171	−5	<0.001	−1405	−639
ABC	3102	1233	2	0.030	352	5851
AB (A-B)	164,942	33,940	4	<0.001	89,318	240,567
AC (A-C)	−158,917	32,925	−4	<0.001	−232,280	−85554
BC(B–C)	170,777	34,999	4	<0.001	92,794	248,759
R^2^	0.948					
R^2^_Adj_	0.902					
**Plasma protection**						
(A)HP	1022	30	33	<0.001	959	1085
(B)EC	230	36	6	<0.001	153	308
(C)PZ	82	30	27	<0.001	758	884
AB	−1633	175	−9	<0.001	−2004	−1263
AC	−1608	170	−9	<0.001	−1968	−1249
BC	−796	153	−5	<0.001	−1120	−472
ABC	9301	1117	8	<0.001	6944	11,659
AB (A-B)	−80526	29,471	−2	0.014	−142,705	−18347
AC (A-C)	73,008	28,595	2	0.020	12,676	133,339
BC(B–C)	−78032	30,390	−2	0.020	−142,151	−13914
R^2^	0.971					
R^2^_Adj_	0.957					
**Cellular antioxidant activity (CAA) in HCT8 cells**						
(A)HP	35	3	10	<0.001	27	42
(B)EC	22	3	6	<0.001	15	29
(C)PZ	161	3	47	<0.001	154	168
AB	−49	16	−3	0.006	−84	−15
AC	−292	16	−17	<0.001	−326	−258
BC	−345	16	−20	<0.001	−379	−310
ABC	300	118	2	0.020	53	547
AB (A-B)	22,527	3258	6	<0.001	15,731	29,323
AC (A-C)	−21655	3160	−6	<0.001	−28248	−15062
BC(B–C)	23,575	3359	7	<0.001	16,567	30,584
R^2^	0.989					
R^2^_Adj_	0.984

For CUPRAC, a similar trend was obtained, where the mean values could be ranked in this order: HP > EC > PZ. Binary combinations (HP-EC and HP-PZ) and the ternary mixture also influenced CUPRAC values. The response plot, as depicted in Fig. 1B, explains 99.8% of data variability and allows for examining the impacts of individual phenolic compounds and their combinations on the CUPRAC values. Regarding HRSA, HP has the highest contribution to the antioxidant potential, followed by EC, and PZ. Binary combinations (AB, AC, BC) did not show synergistic effects on HRSA, but the ternary mixture showed an additive effect. The results can be more effectively visualized through the two-dimensional fitted response plot shown in Fig. 1C. Regarding DPPH, HP had the highest contribution to the free-radical scavenging activity followed by EC and PZ. However, antagonistic effects were clearly shown in binary (HP-EC, HP-PZ), and ternary (HP-EC-PZ) combinations. Fig. 1D visually indicates the effects of phenolic compounds and their combinations on DPPH values. For SRSA, PZ seemed to have the most significant contribution to the antioxidant activity, followed by HP, and EC. In binary (HP and EC) and ternary (HP-EC-PZ) combinations, additive effects were obtained, whereas HP-PZ and EC-PZ showed significant antagonistic effects. The RSM model explained 94.8% of data variability, and Fig. 1E depicts the experimental data in detail. HP, EC, and PZ individually displayed synergistic effects with positive coefficients and significant p-values (p < 0.05). In binary combinations, HP, and EC (AB) showed synergistic effects, while HP and PZ (AC) and EC and PZ (BC) exhibited antagonistic effects. The ternary combination (ABC) was synergistic. Table 2 results for SRSA are better visualized in Fig. 1E.

Several studies have explored how phenolic compounds in plant extracts or pure solutions interact using various antioxidant tests. These studies have noted instances of both synergistic and antagonistic interactions (Baranowska et al., 2021; Capitani et al., 2009; Hajimehdipoor et al., 2014; Skroza et al., 2022). In one study, scientists reported that binary combinations of hydroxybenzoic acids, notably those incorporating gentisic acid, exhibit synergistic antioxidant effects at lower concentrations (100 μM), suggesting potential for enhanced antioxidant activity as assessed through the ferric reducing antioxidant power (FRAP) and oxygen radical absorbance capacity (ORAC) assays (Skroza et al., 2022). However, these effects diminish at higher concentrations (500 μM and 1000 μM). Ternary combinations, particularly protocatechuic, gentisic, and syringic acid, demonstrate significant synergy at lower concentrations, highlighting the complex interplay of compounds and concentration dependencies in optimizing antioxidant efficacy. Also, researchers explored the combined effects of phenolics and flavonoids on antioxidant activity using FRAP assay (Hajimehdipoor et al., 2014). Binary combinations, notably gallic and caffeic acid, demonstrated significant synergistic effects, doubling antioxidant activity. Other binary combinations also exhibited synergy, whereas rutin did not interact with other compounds. However, some ternary combinations showed antagonistic effects, reducing antioxidant activity. They indicated that combining more than two compounds could diminish overall effectiveness. Despite observing reliable synergistic effects in specific ternary combinations, binary combinations remain preferable for maximizing antioxidant activity. Another study discovered possible synergistic blends to boost antioxidant efficacy in food items. Ideal combinations comprised 47% caffeic acid combined with 53% carnosic acid, 67% quercetin, and 33% rutin (Capitani et al., 2009).

The chemical antioxidant activity of phenolic compounds depends on several intrinsic and extrinsic factors: reactional medium's pH, concentration of the free radical/ROS in the medium, reaction time and temperature, and solubility of compounds. Deprotonation and formation of radical anions differ between polyphenols, and the thermodynamics that favor the antioxidant action changes significantly (Klein et al., 2016), including HAT and SET/SPLET mechanisms. In addition, the presence of the sugar moiety significantly contributes to the efficacy of both HP and PZ, while EC lacks this structural component (Fig. S1).

### 3.2 Effect of HP, EC, and PZ on copper-induced plasma oxidation

Lipid peroxidation is a key process linked to oxidative stress, causing damage to polyunsaturated fatty acids (PUFAs) in cell membranes (Halliwell, 2023). The process begins with the initiation reaction, leading to a chain reaction, including the propagation reactions.

To study any antioxidant effects using human plasma as a source of PUFA, HP, EC, PZ, and their binary/ternary combinations were tested. The results (Table 1 and Fig. 1F) show that these compounds could protect human plasma from copper-induced lipid peroxidation, likely by slowing the propagation rate by reacting with chain-propagating peroxyl radicals and ROS generation via a Fenton-like reaction. Results show that the antioxidant activity varied between polyphenols. HP exhibited higher (p < 0.05) antioxidant activity (1022 mg AAE/L) compared to EC (230 mg AAE/L) and PZ (789 mg AAE/L). HP's superior plasma antioxidant protection can be attributed to two hydroxyl groups in the *ortho* position at C-3′ and C-4′ on the aromatic ring B (e.g., catechol system). This characteristic makes HP an efficient radical scavenger. On the other hand, EC, possessing two *ortho* hydroxyls, displayed the lowest human plasma protection, whereas PZ, with two hydroxyls in the *meta* position, showed an intermediate protection of human plasma oxidation.

Table 2 shows the RSM model for plasma protection: 97.1% of data variability was explained, and a significant (p < 0.001) antagonistic effect (HP-EC, HP-PZ, and EC-PZ) was observed. However, the ternary interaction between polyphenols had a synergistic (p < 0.001) antioxidant activity. Flavonoids with additional hydroxyl groups and electron-donating substituents at positions C-5 and C-7 demonstrate inhibiting more lipid peroxidation (Heijnen et al., 2002). This observation could explain the heightened antioxidant activity observed in HP against lipid peroxidation. Polyphenols may act as antioxidants or pro-oxidants depending on the system composition and pH (Cao et al., 1997). Our results show that in this biological system, the number of hydroxyl groups alone cannot be a predictor of antioxidant activity toward lipid peroxidation. Other factors, such as the position of hydroxyl groups, energy ionization, O–H bond dissociation enthalpy, and the nature of substituents (H atom or the presence of a sugar moiety) may also play a central role in the inhibition of lipid peroxidation (Ortega-Moo et al., 2016).

### 3.3 Effects of HP, EC, and PZ on cell growth, proliferation, and cellular antioxidant activity

**Fig. 2:**
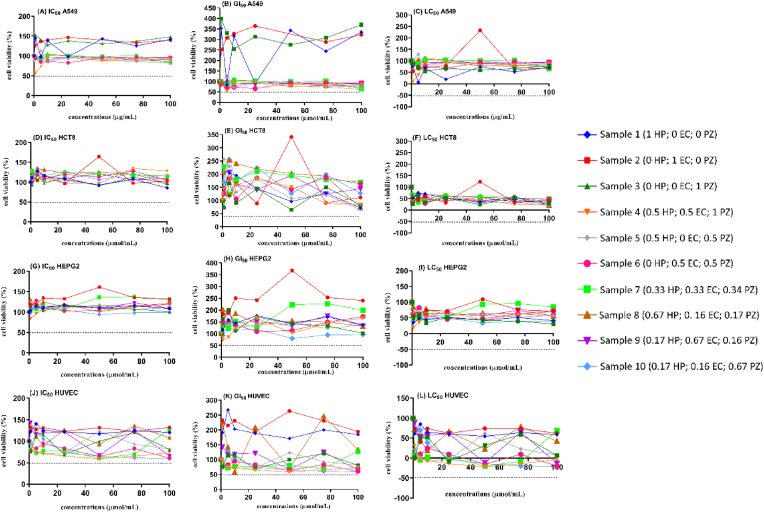
Cell viability and proliferation evaluation of the concentration-dependent effect after 48 h exposure to HP, EC, and PZ compounds in A549 (A, B, and C), HCT8 (D, E, and F), HEPG2 (G, H, and I), and HUVEC (J, K, and L) cell lines. IC_50_ (concentration of the extracts that inhibit cell viability by 50%), GI_50_ (concentration of the extracts that inhibits cell growth by 50%); and LC_50_ (concentrations of the extracts that result in the loss of 50% cells). The numbers between parentheses means the ratio of HP (hyperoside), EC (epicatechin) and PZ (phlorizin).

For the cytotoxicity effect of HP, EC, and PZ against normal and cancer cells, none of the concentrations tested caused cell death, reduced viability, or inhibited cell growth (Fig. 2), showing no selectivity among the cells tested. An evaluation of the impact of HP on yeasts revealed no cytotoxicity at 10–40 mg/L (Gao et al., 2019). In another study, different concentrations of EC (5–20 μM) were assessed, and none of the concentrations selected evoked cell damage or decreased the viability of pancreatic beta cells after 20 h of treatment (Martín et al., 2014). The same result was observed by Wang et al. (2019), which tested PZ treatment at 50, 100, and 150 μg/mL, and no toxicity on HepG2 cells was observed.

**Fig. 3:**
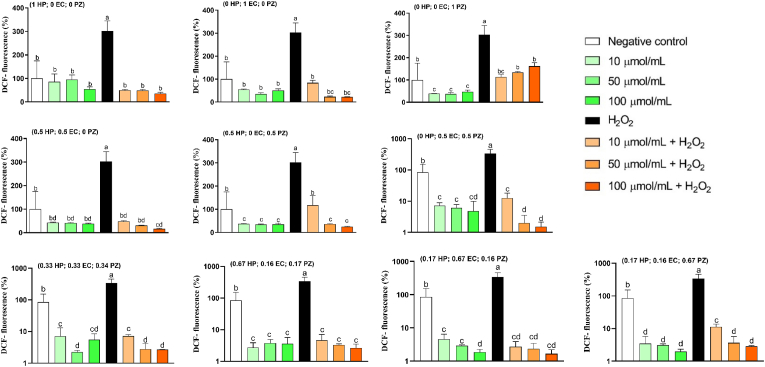
Intracellular ROS-generation in HCT8 cells measurements by spectrofluorimetric. Quantitative data are the mean ± standard deviation (n = 4). Different letters represent statistically significant differences (p ≤ 0.05). Sample identification can be found in Table 1. The numbers between parentheses means the ratio of the flavonoids. HP: hyperoside, EC: epicatechin, PZ: phlorizin.

Regarding the CAA in HCT8 cells (Table 1 and Fig. 3), varying levels of antioxidant activity were observed between polyphenols and their combinations. EC exhibited the highest antioxidant activity (i.e., lowest intracellular ROS generation), followed by HP. Similar results are observed by Martín et al. (2014), who treated Ins-1E cells with 5–20 μM of EC that reduced *t*-BOOH-induced ROS production similar to those observed in control unchallenged cells. HP and EC display the highest cellular antioxidant activity since the ROS levels reached below the cell's basal conditions. This CAA through diverse pathways, including lowering intracellular ROS generation (Gao et al., 2019), enhancing antioxidant defences via the mitogen-activated protein kinase (MAPK)-dependent Kelch-like ECH Homology (ECH)-associated protein 1 (Keap 1)-nuclear factor erythroid 2-related factor 2 (Nrf2)-antioxidant response element (ARE) signaling pathway (Xing et al., 2011), and activating enzymes, such as heme oxygenase-1 (HO-1) through the activation of the Nrf2-extracellular signal-regulated kinase (ERK) signaling pathway (Park et al., 2016). These mechanisms protect cells from oxidative stress by regulating inflammation, autophagy, and apoptosis-related pathways in response to hydrogen peroxide-induced oxidative stress (Feng et al., 2021).

In contrast, PZ demonstrated the lowest antioxidant activity among the individual compounds. In fact, PZ increased intracellular ROS generation at 100 mg/L, showing a significant pro-oxidant behavior. These results contrast with previous research showing that PZ exhibited cellular antioxidant activity through the activation of the activated protein kinase/Nrf2 antioxidative pathway (Yang et al., 2019), the inhibition of ROS production and pro-inflammatory responses (Zhai et al., 2015), as well as the enhancement of cellular antioxidant enzyme activity, such as catalase, superoxide dismutase, and glutathione peroxidase, leading to increased total antioxidant capacity (Liu et al., 2021). However, in HepG2 cells, PZ did not show any significant cellular antioxidant activity compared to the negative control group (Wang et al., 2019). Thus, we demonstrated that PZ had the lowest CAA values and exhibited the lowest chemical antioxidant activity (e.g., FCRC, CUPRAC, HRSA, and DPPH).

In Table 2 and Fig. 1G, the RSM results for the CAA data are presented. HP and EC tended to decrease intracellular ROS generation in H_2_O_2_-treated HCT8 cells, while PZ had the opposite behavior. Binary combinations (HP-EC, HP-PZ, and EC-PZ) showed synergism in decreasing intracellular ROS generation, but the ternary mixture (HP-EC-PZ) seemed to increase the generation of free radicals in HCT8 cells, thus displaying an antagonistic effect. The regression model explained 99% of data variability and can be used for prediction purposes when different flavonoid proportions need to be tested.

Commonly, research subjects cells to extracts abundant in polyphenols rather than isolated substances to assess their impact on inhibiting cell proliferation or combating cancer (Gao et al., 2023; Rufino-Palomares et al., 2022; Teniente et al., 2023). These extracts comprise various compounds, forming an intricate blend that poses challenges in pinpointing synergistic or antagonistic interactions. Thus, the current study applied isolated substances to comprehend the binary and ternary interactions among the selected polyphenols and their effects on cells. Previous reports suggest that combining pure polyphenolics can have synergistic effects against colon cancer, like curcumin with resveratrol (Majumdar et al., 2009), EC with epigallocatechin-gallate (Shimizu et al., 2005), or 5-fluorouracil with curcumin (Du et al., 2005). Also, the effects of combining phenolic compounds, such as delphinidin-3-rutinoside and EC, and chlorogenic acid on gastric and intestinal cancer cells were reported (Miladinovic et al., 2023). Certain combinations like delphinidin-3-rutinoside with chlorogenic acid and EC in gastric cancer cells showed synergistic effects at lower doses (e.g., until IC_25_). Conversely, in intestinal cancer cells, antagonism was noted at lower doses across all combinations, with varying degrees of synergism observed at higher doses (e.g., >IC_80_). The study emphasized the potential benefits of synergistic combinations in reducing dosage, offering advantages for cancer treatment strategies (Miladinovic et al., 2023).

### 3.4 Correlation analysis

The statistical significance between antioxidant methods, especially CAA and the plasma protection, was assay-dependent: CAA was significantly correlated with CUPRAC (r = −0.575, p = 0.003) and SRSA (r = 0.6995, p < 0.001) but not with HRSA (r = −0.241, p = 0.246), FCRC (r = −0.211, p = 0.312) and DPPH (r = −0.340, p = 0.096). The plasma protection against copper-induced oxidation did not correlate (p > 0.05) with any chemical and CAA assay. These findings underscore the complexity of the relationship between chemical antioxidant activity and its relevance to the oxidative and antioxidative processes in human cells and plasma. Therefore, it is essential to recognize that compounds exhibiting “high” antioxidant activity in chemical assays may not necessarily translate into apparent protective effects on cells within a complex biological system, and vice versa (Granato, 2023).

Using RAW 264.7 macrophages and several chemical antioxidant activity methods, it was found that the oxygen radical absorbance capacity (ORAC), FCRC, and ferric-reducing antioxidant power (FRAP) were significantly associated with the reduction in the activation of NF-κB, concluding that screening assays are simple and high throughput options (de Camargo et al., 2019). Indeed, factors such as experimental conditions and the specific cellular or chemical context can significantly influence how compounds behave (Granato, 2023; Pasqualetti et al., 2021). For example, various conditions are used in chemical antioxidant assays. These include varying pH levels (ranging from 3 to 12), temperatures (typically between 25 and 37 °C), reaction times (ranging from 5 min to 2 h), and the use of probes and radicals like DPPH, which are not commonly found in biological systems (Apak et al., 2022; Cruz et al., 2024). However, biological systems are conducted at physiological pH and temperature, focusing on accounting for metabolism and intracellular transportation. Hence, the differences in the results of some chemical assays in this study constitute a limitation of these assays in examining molecules with antioxidant properties. This limitation primarily arises from their failure to consider relevant parameters present in CAA (Pasqualetti et al., 2021). Also, it is reported that the highest level of biological activity doesn't consistently correspond to higher values of factors indicating potential bioavailability and bioaccessibility (González-Sarrías et al., 2015). When comparing the antioxidant activity of phenolic compounds, it can be noted that the composition of hydroxyl groups, the presence of sugar moieties, molecular weight, and solubility in the reactional medium are primarily responsible for generating antioxidant potential via SET/SPLET, HAT, and metal chelation mechanisms of action. However, the significance of individual compounds and the interactions among phenolic compounds are also essential factors (Świeca et al., 2017). Approaching a scientific evidence-based answer to “Should we ban chemical antioxidant screening methods?” the answer is “no” as experimental results show their importance in assessing the antioxidant potential of pure compounds and bioactive-rich extracts. It is critical to emphasize that chemical measures of antioxidant activity do not faithfully represent the conditions within cells, but some assays show prominent usability in understanding complex biological systems. As a result, it is imperative to employ both *in vitro* chemical and human cell models in antioxidant activity assessment.

## 4 Conclusion

In this study, HP exhibited superior antioxidant activity in various assays, particularly FCRC, CUPRAC, HRSA, and DPPH, while PZ excelled in SRSA. Binary combinations of these compounds generally showed intermediate results in the tested antioxidant activities, and synergism between the three polyphenols was not apparent in most assays. When combined in a specific ratio, polyphenols demonstrated a synergistic effect with the potential to enhance plasma protection against oxidative stress. HP and EC effectively alleviated oxidative stress induced by H_2_O_2_ in HCT8 cells, but PZ showed pro-oxidant effects. It was found that the antioxidant activity in chemical assays did not necessarily translate into protective effects in a biological system and vice versa. Thus, the combined use of chemical and biological assays to assess the antioxidant potential of food-derived materials is incentivized.

## CRediT authorship contribution statement

**Nima Mohammadi:** Methodology, Formal analysis, Investigation, Writing – original draft. **Amanda dos Santos Lima:** Methodology, Formal analysis, Investigation, Writing – original draft. **Luciana Azevedo:** Project administration, Funding acquisition, Writing – review & editing. **Daniel Granato:** Conceptualization, Project administration, Funding acquisition, Writing – original draft, Writing – review & editing.

## Declaration of competing interest

The authors declare that they have no known competing financial interests or personal relationships that could have appeared to influence the work reported in this paper.

## Data Availability

Data will be made available on request.

## References

[bib1] Apak R., Calokerinos A., Gorinstein S., Segundo M.A., Hibbert D.B., Gülçin I., Demirci Çekiç S., Güçlü K., Özyürek M., Çelik S.E., Magalhães L.M., Arancibia-Avila P. (2022). Methods to evaluate the scavenging activity of antioxidants toward reactive oxygen and nitrogen species (IUPAC Technical Report). Pure Appl. Chem..

[bib2] Baranowska M., Koziara Z., Suliborska K., Chrzanowski W., Wormstone M., Namieśnik J., Bartoszek A. (2021). Interactions between polyphenolic antioxidants quercetin and naringenin dictate the distinctive redox-related chemical and biological behaviour of their mixtures. Sci. Rep..

[bib3] Caliskan R., Sari S.P., Altinbasak B.B., Dinc H.O., Balekoglu A., Issa G., Mayda P.Y. (2022). Bioactive components and antioxidant and antimicrobial activities of Rhus coriaria, a sumac species found in Turkey. Bezmialem Sci.

[bib4] Cao G., Sofic E., Prior R.L. (1997). Antioxidant and prooxidant behavior of flavonoids: structure-activity relationships. Free Radic. Biol. Med..

[bib5] Capitani C.D., Carvalho A.C.L., Botelho P.B., Carrapeiro M.M., Castro I.A. (2009). Synergism on antioxidant activity between natural compounds optimized by response surface methodology. Eur. J. Lipid Sci. Technol..

[bib6] Chen Y., Xiao H., Zheng J., Liang G. (2015). Structure-thermodynamics-antioxidant activity relationships of selected natural phenolic acids and derivatives: an experimental and theoretical evaluation. PLoS One.

[bib7] Corrigan H., Dunne A., Purcell N., Guo Y., Wang K., Xuan H., Granato D. (2023). Conceptual functional-by-design optimisation of the antioxidant capacity of trans-resveratrol, quercetin, and chlorogenic acid: application in a functional tea. Food Chem..

[bib8] Cruz T.M., Lima A.dos S., Silva A.O., Mohammadi N., Zhang L., Azevedo L., Marques M.B., Granato D. (2024). High-throughput synchronous erythrocyte cellular antioxidant activity and protection screening of phenolic-rich extracts: protocol validation and applications. Food Chem..

[bib9] Cui S., Ma X., Wang X., Zhang T.A., Hu J., Tsang Y.F., Gao M.T. (2019). Phenolic acids derived from rice straw generate peroxides which reduce the viability of Staphylococcus aureus cells in biofilm. Ind. Crops Prod..

[bib10] de Camargo A.C., Biasoto A.C.T., Schwember A.R., Granato D., Rasera G.B., Franchin M., Rosalen P.L., Alencar S.M., Shahidi F. (2019). Should we ban total phenolics and antioxidant screening methods? The link between antioxidant potential and activation of NF-κB using phenolic compounds from grape by-products. Food Chem..

[bib11] do Carmo M.A.V., Pressete C.G., Marques M.J., Granato D., Azevedo L. (2018). Polyphenols as potential antiproliferative agents: scientific trends. Curr. Opin. Food Sci..

[bib12] Du B., Jiang L., Xia Q., Zhong L. (2005). Synergistic inhibitory effects of curcumin and 5-fluorouracil on the growth of the human colon cancer cell line HT-29. Chemotherapy.

[bib13] Erskine E., Gültekin Subaşl B., Vahapoglu B., Capanoglu E. (2022). Coffee phenolics and their interaction with other food phenolics: antagonistic and synergistic effects. ACS Omega.

[bib14] Escher G.B., Santos J.S., Rosso N.D., Marques M.B., Azevedo L., do Carmo M.A.V., Daguer H., Molognoni L., Prado-Silva L. do, Sant'Ana A.S., da Silva M.C., Granato D. (2018). Chemical study, antioxidant, anti-hypertensive, and cytotoxic/cytoprotective activities of Centaurea cyanus L. petals aqueous extract. Food Chem. Toxicol..

[bib15] Feng Y., Wang D., Wang Q., Li Z., Yang S.-L., Feng Y.-L., Luo T., Li Y. (2021). Protective effects and mechanism of hyperoside in PC12 cells against oxidative stress injury induced by hydrogen peroxide. Nat. Prod. Commun..

[bib16] Gao N., Si X., Han W., Gong E., Shu C., Tian J., Wang Y., Zhang J., Li Binxu, Li Bin (2023). The contribution of different polyphenol compositions from chokeberry produced in China to cellular antioxidant and antiproliferative activities. Food Sci. Hum. Wellness.

[bib58] Fidelis M., Santos J.S., Escher G.B., Carmo M.A.V., Azevedo L., Silva M.C., Granato D. (2018). *In vitro* antioxidant and antihypertensive compounds from camu-camu (*Myrciaria dubia* McVaugh, Myrtaceae) seed coat: a multivariate structure-activity study. Food Chem. Toxicol..

[bib17] Gao Y., Fang L., Wang X., Lan R., Wang M., Du G., Guan W., Liu J., Brennan M., Guo H., Brennan C., Zhao H. (2019). Antioxidant activity evaluation of dietary flavonoid hyperoside using Saccharomyces cerevisiae as a model. Molecules.

[bib18] Gonzalez-Alfonso J.L., Ubiparip Z., Jimenez-Ortega E., Poveda A., Alonso C., Coderch L., Jimenez-Barbero J., Sanz-Aparicio J., Ballesteros A.O., Desmet T., Plou F.J. (2021). Enzymatic synthesis of phloretin α-glucosides using a sucrose phosphorylase mutant and its effect on solubility, antioxidant properties and skin absorption. Adv. Synth. Catal..

[bib19] González-Sarrías A., García-Villalba R., Núñez-Sánchez M.Á., Tomé-Carneiro J., Zafrilla P., Mulero J., Tomás-Barberán F.A., Espín J.C. (2015). Identifying the limits for ellagic acid bioavailability: a crossover pharmacokinetic study in healthy volunteers after consumption of pomegranate extracts. J. Funct.Foods.

[bib20] Granato D. (2023). Next-generation analytical platforms for antioxidant capacity assessment: the urge for realistic and physiologically relevant methods. Biomed. Pharmacother..

[bib21] Granato D., Shahidi F., Wrolstad R., Kilmartin P., Melton L.D., Hidalgo F.J., Miyashita K., Camp J. van, Alasalvar C., Ismail A.B., Elmore S., Birch G.G., Charalampopoulos D., Astley S.B., Pegg R., Zhou P., Finglas P. (2018). Antioxidant activity, total phenolics and flavonoids contents: should we ban in vitro screening methods?. Food Chem..

[bib22] Hajimehdipoor H., Shahrestani R., Shekarchi M. (2014). Investigating the synergistic antioxidant effects of some flavonoid and phenolic compounds. Res. J. Pharmacogn..

[bib23] Halliwell B. (2023). Understanding mechanisms of antioxidant action in health and disease. Nat. Rev. Mol. Cell Biol..

[bib60] Heijnen C.G.M., Haenen G.R.M.M., Minou Oostveen R., Stalpers E.M., Bast A. (2002). Protection of flavonoids against lipid peroxidation: The structure activity relationship revisited. Free Radic. Res..

[bib24] Jacobs D.R., Temple N.J., Temple N.J., Wilson T., Jacobs Jr D.R. (2012). Nutritional Health: Strategies for Disease Prevention.

[bib25] Jang E. (2022). Hyperoside as a potential natural product targeting oxidative stress in liver diseases. Antioxidants.

[bib26] Jug U., Naumoska K., Vovk I. (2021). (−)-Epicatechin—an important contributor to the antioxidant activity of Japanese knotweed rhizome bark extract as determined by antioxidant activity-guided fractionation. Antioxidants.

[bib27] Klein E., Rimarčík J., Senajová E., Vagánek A., Lengyel J. (2016). Deprotonation of flavonoids severely alters the thermodynamics of the hydrogen atom transfer. Comput. Theor. Chem..

[bib28] Leopoldini M., Russo N., Toscano M. (2011). The molecular basis of working mechanism of natural polyphenolic antioxidants. Food Chem..

[bib29] Liu Yaojie, Liu Ying, Guo Y., Xu L., Wang H. (2021). Phlorizin exerts potent effects against aging induced by d-galactose in mice and PC12 cells. Food Funct..

[bib30] Majumdar A.P.N., Banerjee S., Nautiyal J., Patel B.B., Patel V., Du J., Yu Y., Elliott A.A., Levi E., Sarkar F.H. (2009). Curcumin synergizes with resveratrol to inhibit colon cancer. Nutr. Cancer.

[bib31] Martín M.Á., Fernández-Millán E., Ramos S., Bravo L., Goya L. (2014). Cocoa flavonoid epicatechin protects pancreatic beta cell viability and function against oxidative stress. Mol. Nutr. Food Res..

[bib32] Mendes R.A., e Silva B.L.S., Takeara R., Freitas R.G., Brown A., de Souza G.L.C. (2018). Probing the antioxidant potential of phloretin and phlorizin through a computational investigation. J. Mol. Model..

[bib33] Miladinovic B., Faria M.Â., Ribeiro M., Sobral M.M.C., Ferreira I.M. (2023). Delphinidin-3-rutinoside from blackcurrant berries (ribes nigrum): in vitro antiproliferative activity and interactions with other phenolic compounds. Molecules.

[bib34] Mohammadi N., Guo Y., Wang K., Granato D. (2024). Macroporous resin purification of phenolics from Irish apple pomace: chemical characterization, and cellular antioxidant and anti-inflammatory activities. Food Chem..

[bib35] Ongay K.K., Granato D., Barreto G.E. (2023). Comparison of antioxidant capacity and network pharmacology of phloretin and phlorizin against neuroinflammation in traumatic brain injury. Molecules.

[bib36] Ortega-Moo C., Garza J., Vargas R. (2016). The substituent effect on the antioxidant capacity of catechols and resorcinols. Theor. Chem. Acc..

[bib37] Park J.Y., Han X., Piao M.J., Oh M.C., Fernando P.M.D.J., Kang K.A., Ryu Y.S., Jung U., Kim I.G., Hyun J.W. (2016). Hyperoside induces endogenous antioxidant system to alleviate oxidative stress. J. Cancer Prev..

[bib38] Pasqualetti V., Locato V., Fanali C., Mulinacci N., Cimini S., Morgia A.M., Pasqua G., De Gara L. (2021). Comparison between in vitro chemical and ex vivo biological assays to evaluate antioxidant capacity of botanical extracts. Antioxidants.

[bib39] Peyrat-Maillard M.N., Cuvelier M.E., Berset C. (2003). Antioxidant activity of phenolic compounds in 2,2′-azobis (2-amidinopropane) dihydrochloride (AAPH)-Induced oxidation: synergistic and antagonistic effects. JAOCS, J. Am. Oil Chem. Soc..

[bib40] Rezk B.M., Haenen G.R.M.M., Van der Vijgh W.J.F., Bast A. (2002). The antioxidant activity of phloretin: the disclosure of a new antioxidant pharmacophore in flavonoids. Biochem. Biophys. Res. Commun..

[bib41] Rufino-Palomares E.E., Pérez-Jiménez A., García-Salguero L., Mokhtari K., Reyes-Zurita F.J., Peragón-Sánchez J., Lupiáñez J.A. (2022). Nutraceutical role of polyphenols and triterpenes present in the extracts of fruits and leaves of olea europaea as antioxidants, anti-infectives and anticancer agents on healthy growth. Molecules.

[bib42] Sang S., Liao C.H., Pan M.H., Rosen R.T., Lin-Shiau S.Y., Lin J.K., Ho C.T. (2002). Chemical studies on antioxidant mechanism of garcinol: analysis of radical reaction products of garcinol with peroxyl radicals and their antitumor activities. Tetrahedron.

[bib43] Shimizu M., Deguchi A., Lim J.T.E., Moriwaki H., Kopelovich L., Weinstein I.B. (2005). (-)-Epigallocatechin gallate and polyphenon E inhibit growth and activation of the epidermal growth factor receptor and human epidermal growth factor receptor-2 signaling pathways in human colon cancer cells. Clin. Cancer Res..

[bib44] Skroza D., Šimat V., Vrdoljak L., Jolić N., Skelin A., Čagalj M., Frleta R., Generalić Mekinić I. (2022). Investigation of antioxidant synergisms and antagonisms among phenolic acids in the model matrices using FRAP and ORAC methods. Antioxidants.

[bib45] Sukito A., Tachibana S. (2014). Isolation of hyperoside and isoquercitrin from camellia sasanqua as antioxidant agents. Pakistan J. Biol. Sci..

[bib46] Świeca M., Gawlik-Dziki U., Dziki D., Baraniak B. (2017). Wheat bread enriched with green coffee – in vitro bioaccessibility and bioavailability of phenolics and antioxidant activity. Food Chem..

[bib47] Tejero I., González-García N., González-Lafont A., Lluch J.M. (2007). Tunneling in green tea: understanding the antioxidant activity of catechol-containing compounds. A variational transition-state theory study. J. Am. Chem. Soc..

[bib48] Teniente S.L., Flores-Gallegos A.C., Esparza-González S.C., Campos-Múzquiz L.G., Nery-Flores S.D., Rodríguez-Herrera R. (2023). Anticancer effect of pomegranate peel polyphenols against cervical cancer. Antioxidants.

[bib49] Uchida K., Ogawa K., Yanase E. (2016). Structure determination of novel oxidation products from epicatechin: thearubigin-like molecules. Molecules.

[bib50] Vagánek A., Rimarčík J., Dropková K., Lengyel J., Klein E. (2014). Reaction enthalpies of OH bonds splitting-off in flavonoids: the role of non-polar and polar solvent. Comput. Theor. Chem..

[bib51] Wang Hao, Cheng J., Wang Huali, Wang M., Zhao J., Wu Z. (2019). Protective effect of apple phlorizin on hydrogen peroxide-induced cell damage in HepG2 cells. J. Food Biochem..

[bib52] Xing H.Y., Liu Y., Chen J.H., Sun F.J., Shi H.Q., Xia P.Y. (2011). Hyperoside attenuates hydrogen peroxide-induced L02 cell damage via MAPK-dependent Keap 1-Nrf2-ARE signaling pathway. Biochem. Biophys. Res. Commun..

[bib53] Xu S., Chen S., Xia W., Sui H., Fu X. (2022). Hyperoside: a review of its structure, synthesis, pharmacology, pharmacokinetics and toxicity. Molecules.

[bib54] Xu Y.Q., Gao Y., Granato D. (2021). Effects of epigallocatechin gallate, epigallocatechin and epicatechin gallate on the chemical and cell-based antioxidant activity, sensory properties, and cytotoxicity of a catechin-free model beverage. Food Chem..

[bib55] Xue Y., Zheng Y., Zhang L., Wu W., Yu D., Liu Y. (2013). Theoretical study on the antioxidant properties of 2′- hydroxychalcones: H-atom vs. electron transfer mechanism. J. Mol. Model..

[bib56] Yang Q., Han L., Li J., Xu H., Liu X., Wang X., Pan C., Lei C., Chen H., Lan X. (2019). Activation of Nrf2 by phloretin attenuates palmitic acid-induced endothelial cell oxidative stress via AMPK-dependent signaling. J. Agric. Food Chem..

[bib57] Zhai Y., Dang Y., Gao W., Zhang Y., Xu P., Gu J., Ye X. (2015). P38 and JNK signal pathways are involved in the regulation of phlorizin against UVB-induced skin damage. Exp. Dermatol..

[bib59] Zhang Q.A., Wang X., Song Y., Fan X.H., García-Martín J.F. (2016). Optimization of pyrogallol autoxidation conditions and its application in evaluation of superoxide anion radical scavenging capacity for four antioxidants. J. AOAC Int..

