# Supplementary figures and images for: Bridging the gap in antioxidant activity of flavonoids: Correlating the oxidation of human plasma with chemical and cellular assays
Source: Curr Res Food Sci. 2024 Mar 13;8:100714. doi: 10.1016/j.crfs.2024.100714 (PMC10965461; doi:10.1016/j.crfs.2024.100714)

| A |  | B |  |
| --- | --- | --- | --- |
|  | 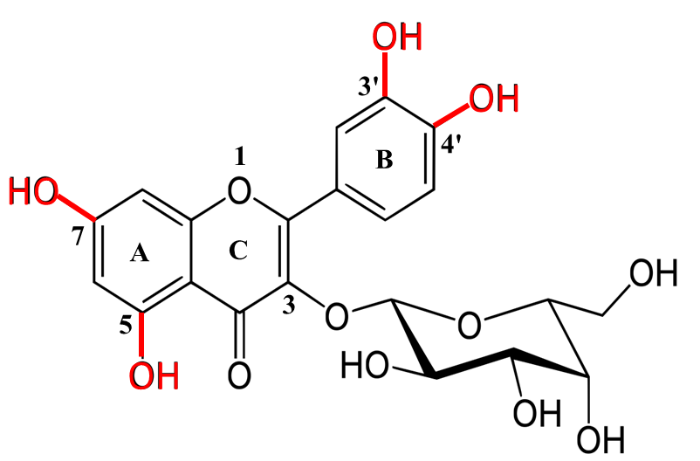 |  | 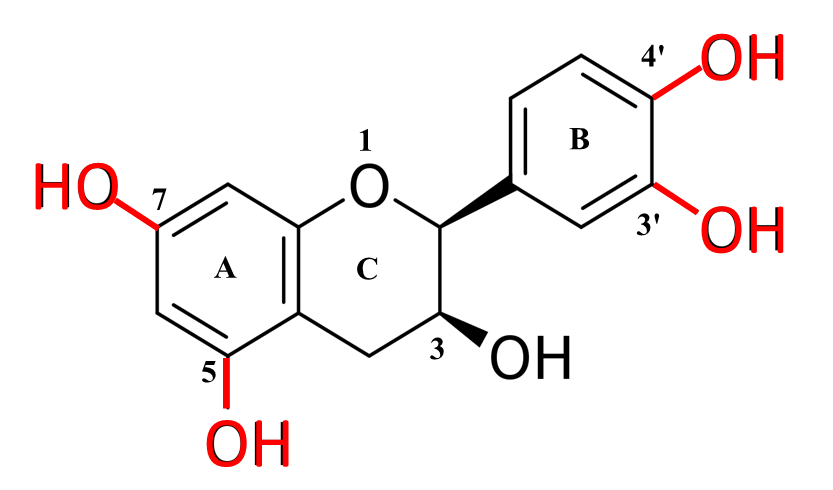 |
| C | 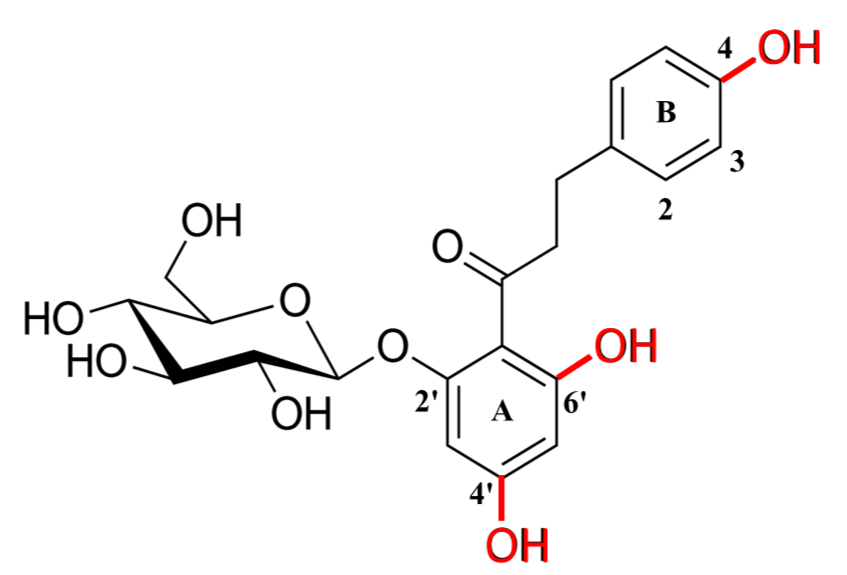 | | |

**Fig. S1.** Chemical structures of hyperoside (A), epicatechin (B), and phlorizin (C).

Supplement: Multimedia component 1 [file mmc1.docx]
